# Correlations between hemodynamics and radiomic features in thrombosed intracranial aneurysms

**DOI:** 10.1007/s00234-025-03730-x

**Published:** 2025-08-18

**Authors:** Zonghan Lyu, Andres Gudino, Navami Shenoy, Carlos Dier, Elena Sagues, Jingfeng Jiang, Edgar A Samaniego

**Affiliations:** 1 Michigan Technological University, Houghton, United States; 2 University of Iowa, Iowa City, United States

**Keywords:** Intracranial aneurysms, Intrasaccular thrombosis, High-resolution magnetic resonance imaging, Aneurysm wall enhancement, Radiomics analysis, Computational fluid dynamics

## Abstract

**Purpose:**

Evaluating intracranial aneurysm (IA) rupture risk is essential for guiding management. Although intrasaccular thrombosis (IST) is less common, it can contribute to aneurysm growth, mass effect, and rupture. Aneurysm wall enhancement (AWE) on high-resolution MRI (HR-MRI) offers valuable insight into IST and IA progression. Using radiomics, we extracted spatial information of the aneurysm wall to characterize AWE. This study aimed to explore correlations between radiomics-based AWE profiles and gross hemodynamic parameters, integrating imaging and flow dynamics to better understand IST.

**Methods:**

Radiomic analysis was conducted on a cohort of 3T HR-MRI scans from IA with IST. Three-dimensional vascular reconstructions and CFD simulations were conducted to quantify hemodynamic parameters. Spearman’s correlation was performed to correlate aneurysm morphology, AWE patterns, and aneurysmal hemodynamic characteristics.

**Results:**

A total of 37 thrombosed IAs were included in the analysis, comprising 22 fusiform (59.5%) and 15 saccular (40.5%) aneurysms. Six AWE RFs demonstrated strong correlations with aneurysm volume and surface area (ρ > 0.7 for both). Ten AWE RFs were highly correlated with flow vortex parameters (ρ > 0.7), and one showed a strong correlation with wall shear stress (WSS)-related metrics (ρ > 0.7). In the subset of saccular IAs, 20 AWE RFs were strongly associated with WSS-related metrics. In contrast, fusiform IAs showed stronger correlations between AWE RFs and vortex core characteristics. These findings suggest that elevated AWE is closely associated with regions of high oscillatory shear index and unstable flow vortices, indicating a potential link between wall enhancement and disturbed intra-aneurysmal hemodynamics.

Conclusions

Stagnant flow may promote degenerative remodeling of the aneurysm wall and IST. A combined spatiotemporal analysis of hemodynamic parameters and AWE patterns provide information about underlying biological processes of IAs, including the development of IST.

**Supplementary Information:**

The online version contains supplementary material available at 10.1007/s00234-025-03730-x.

## Introduction

Thrombosed intracranial aneurysms (IAs) represent an uncommon subset of aneurysms, often referred to as tumorous aneurysms due to their large size [1]. Contrary to common belief, these aneurysms have the potential to grow and rupture [1, 2]. However, the pathogenesis of thrombosis in these aneurysms remains controversial. Thrombosed IAs may harbor a phenomenon known as intrasaccular thrombosis (IST). At the same time, another proposed mechanism suggests that intramural hemorrhages within the aneurysm wall could lead to thrombosis, representing an intramural thrombus that may eventually contribute to aneurysm growth [3, 4]. These wall-related processes can coexist with IST and may indicate an underlying dissecting aneurysm rather than a purely saccular pathology. Therefore, in some cases, it is impossible to determine the exact origin of the thrombosis, and a better understanding of IST could provide valuable insights into the behavior of thrombosed IAs.

Radiomics analysis has been used in high-resolution magnetic resonance imaging (HR-MRI) to stratify rupture risk for IAs based on signal intensity (SI) characteristics of the aneurysm wall [5, 6]. Radiomics enables the extraction of a comprehensive set of quantitative features—radiomic features (RFs)—that capture detailed information about aneurysm wall characteristics, particularly aneurysm wall enhancement (AWE) [7]. Other computational tools, such as computational fluid dynamics (CFDs), can be used to study IAs further, as aneurysmal hemodynamics play a crucial role in the natural history of IAs [8, 9]. 

To this end, we aimed at analyzing one of the largest cohorts of aneurysms with IST with radiomic-derived AWE data and CFDs. More specifically, our primary objective was to examine the potential relationship between AWE and comprehensive hemodynamic features, including near-wall and intra-aneurysmal flow patterns. In addition to evaluating wall shear stress (WSS) and its derivatives, gross aneurysmal hemodynamics were characterized through vortex core analysis, which quantifies swirling flow patterns using time-resolved 3D velocity fields [10, 11]. These swirling vortices and eddies, hallmarks of aneurysmal flow, were hypothesized to influence thrombus formation and AWE signal characteristics [11–13]. 

The results of this study will advance our understanding of how complex intra-aneurysmal flow behaviors correlate with radiomic signatures of AWE, potentially enhancing the interpretation of imaging findings in thrombosed IAs.

## Methods and Materials

**Fig. 1:**
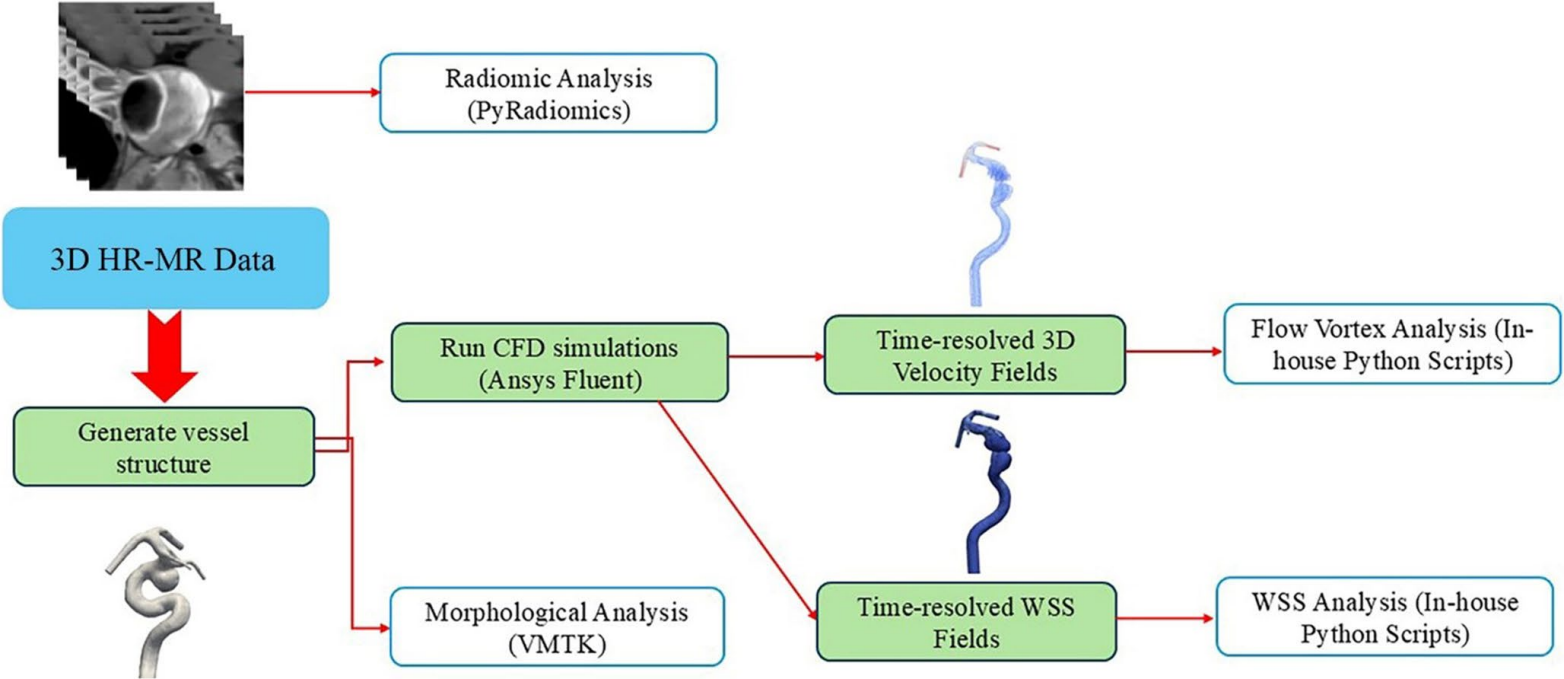
A diagram showing the overall workflow of our data analysis

The workflow involving computational hemodynamic analysis, morphological analysis, and radiomic analysis is schematically summarized in Fig. 1.

### Image Data Acquisition

The institutional review boards at XXX and XXX approved our study protocol as a secondary analysis of existing data. Thus, informed consent was not required.

Thrombosed IAs were identified from an internal database at XXX. All imaging data were acquired using HR-MRI protocol, with a 3 T scanner (Magentom Skyra, SIEMENS) between May 2018 and December 2024. As part of the imaging protocol, T1-weighted (T1) and T1 + Gadolinium (T1 + Gd) sequences were obtained. T1 + Gd images were acquired 5 min after administering 0.1 mmol/Kg gadolinium-based contrast agent (Gadavist, Bayer Pharmaceuticals, Whippany, NJ; Supplementary Material). Images were isotropically resampled and spatially registered following a previously published protocol [14]. The analysis included patients with saccular and fusiform IAs with evidence of IST. Dissecting aneurysms may have been included in the analysis, as in some instances it is impossible to determine the exact location of the thrombus: intramural versus intrasaccular, despite the typical onion-skin appearance of the multilayered thrombus wall of dissecting aneurysms [2]. Demographic information was retrieved from electronic medical records.

### Model Creation and Morphological Analysis

To perform three-dimensional (3D) segmentation, an open-source processing software package (3D Slicer, version 5.6.1,https://www.slicer.org/) was used to mask the extent of each IA using T1 and T1 + Gd images [15]. Specifically, the segmentation process involved delineating the aneurysm and its parent vessels in T1 and T1 + Gd images. Digital subtraction angiography (DSA), magnetic resonance angiography (MRA) or computed tomography angiography (CTA) were used for quality control of the aneurysm boundaries.

Segmented masks in 3D Slicer were converted to a triangulated surface representing the IA vessel geometry. Then, a stereolithography (STL) file was generated for each IA model; each STL file was imported into a commercial software package (3-Matic V.16, Materialise Inc. Leuven, Belgium) for surface quality enhancement. Following a published method [16], an IA sac was first isolated from each triangulated vessel surface (i.e., the vessel model mentioned above). Then, aneurysm volume, aneurysm height, aneurysm width (i.e., maximum width along the direction that is perpendicular to the flow entering the aneurysm), aspect ratio (height/width), parent vessel diameter, and size ratio were calculated.

Other details of the morphological analysis are similar to those used in other publications (e.g [17–19]).,, and are provided in Supplementary Materials for completeness. Morphological metrics were computed using in-house Python scripts (Version 3.8) derived from Visualization ToolKit (VTK, Kitware, NY, USA).

### CFD Simulations

CFD simulations were performed for each IA, following published protocols [20–22], as shown in Fig. 1. Similar CFD simulation protocols have been cross-verified against phase-contrast magnetic resonance angiography (PC-MRA) and ultrasound Doppler velocimetry for aneurysmal flow quantification [23–27].

Processed vessel geometries (see Model Creation Section above) were augmented with cylindrical flow extensions (minimum length: 10× vessel diameter) at all inlets/outlets using the Vascular Modelling Toolkit (VMTK v1.4, www.vmtk.org) to minimize boundary effects. Then, TetGen (Version 1.4.2) was used to generate unstructured 3D tetrahedral (volumetric) meshes for subsequent CFD simulations. All meshes contain five boundary layers with around 0.5–1.0 million elements. A mesh sensitivity analysis was conducted, and we concluded that appropriate mesh density was achieved.

The generated volumetric meshes were processed to solve transient Navier–Stokes equations using FLUENT (Version 21, Ansys Inc., PA, USA). Blood was modeled as an incompressible, laminar, Newtonian fluid with a dynamic viscosity of 0.004 Pa·s and a mass density of 1040 kg/m^3^. All arterial walls were assumed to be rigid, with a no-slip boundary condition.

Suited pulsatile flow (rate) waveforms were implemented as the inlet boundary condition, depending on the anatomical locations of selected aneurysms (e.g., from MR flow measurement in [28]). Zero-pressure boundary conditions were prescribed to all outlets. CFD simulations were performed for four cardiac cycles with 1000 (time) steps per cardiac cycle (0.001 s per time step). Twenty (20) sets of computed WSS and velocity data, temporally (equally) sampled from the last cardiac cycle, were used for the subsequent hemodynamic analysis.

### Hemodynamic Analysis

Once CFD simulations were completed, the calculated WSS and flow fields became available for further analysis. At each point on the aneurysm surface, the WSS vector $\:\tau\:=\left({\tau\:}_{x},{\tau\:}_{y},{\tau\:}_{z}\right)$ was calculated by the Ansys Fluent. These vectors were averaged over 20 time points during the final cardiac cycle to obtain the time-averaged WSS (TAWSS), calculated as follows:

1 $$\:\frac{1}{T}\underset{0}{\overset{T}{\int\:}}\left|\tau\:\right|dt$$

where T is the duration of a simulated cardiac cycle and $\:\left|\tau\:\right|$ stands for the magnitude of the WSS vector.

Using the generated TAWSS field, the endothelial cell activation potential (ECAP), which is linked to the potential of thrombus initiation [29] and the relative residence time (RRT) [30] can be computed for each point on the aneurysm sac:

2 $$\:\frac{OSI}{TWSS}$$

3 $$\:\frac{1}{\left(1-2*OSI\right)*TWSS}$$

Similarly, the oscillatory shear index (OSI) for each point on the aneurysm sac was calculated by determining the variation of the WSS vector over the cardiac cycle, as follows: [31]:

4 $$\:\frac{1}{2}\left(1-\frac{\underset{0}{\overset{T}{\int\:}}\tau\:\:dt}{\underset{0}{\overset{T}{\int\:}}\left|\tau\:\right|dt}\right)$$

In addition to WSS derived parameters, vortex analysis was performed to extract the vortex volume(VV) and number of vortex cores (NOC) to represent the gross aneurysmal hemodynamics.

**Fig. 2:**
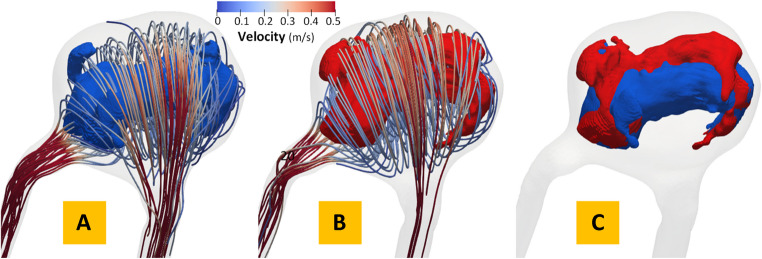
An example showing complex flow disturbance in a saccular IA with a wide neck The degree of volume overlap (DVO) of flow vortex cores between two phases of a cardiac cycle: (**A**) the i^th^ time-step (blue), (**B**) the (i + 1)^th^ time-step (red), and (**C**) minimal flow vortex core overlaps indicating significant flow complexity. Velocity streamlines show the overall flow pattern at each phase

As shown in Fig. 2, swirling flow eddies (i.e., recirculation zones) are present in the aneurysmal sac. Typically, flow eddies shift, break, and merge in space over a cardiac cycle. Using a published method [11, 32, 33], we first masked out the flow vortex core regions for each phase of the cardiac cycle (see the blue and red surfaces in Fig. 2A and B). Then, we calculated the degree of overlap (DVO) defined as the overlap ratio between the flow vortex core regions at two adjacent phases of the cardiac cycle (see Fig. 2C). Visualization of flow vortex cores over a cardiac cycle overlaid with time-resolved streamlines of the IA is shown in Figs. 2A-B.

Sections 2 and 3 of Supplementary Materials include more WSS and flow vortex metrics details. These metrics were calculated using in-house Python scripts (Version 3.8) derived from the open-source VTK/VMTK (Version 1.4) software package.

### Radiomic Analysis of VWI Data

**Fig. 3:**
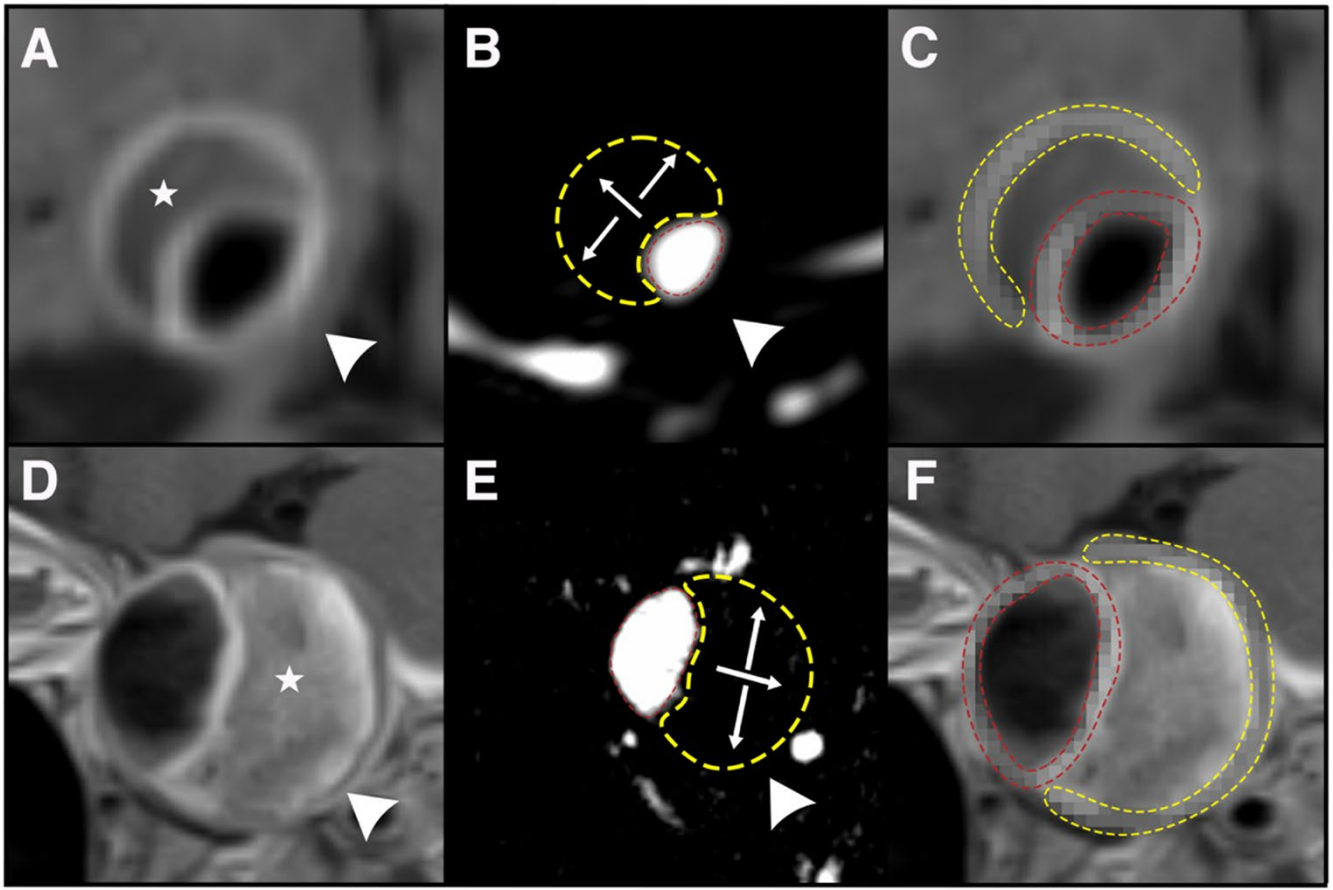
A terminal internal carotid artery (ICA) saccular aneurysm (**A**) with intrasaccular thrombosis (white star) is identified (arrowhead) in high-resolution magnetic resonance imaging (HR-MRI). Likewise, a (**D**) cavernous ICA fusiform aneurysm with IST (white star) is visualized in HR-MRI (arrowhead). (**B** & **E**) The boundaries of the thrombus (white arrows, yellow circle) are then identified on magnetic resonance imaging (MRA). (**C**, **F**) The wall next to the thrombus (yellow circle) and free of thrombus (red circle) is isolated, and radiomics are extracted

In 3D slicer, the thrombus boundaries were identified and isolated. A senior investigator (XXX) adjudicated the boundaries of the thrombus using DSA and MRA. HR-MRI was then used to isolate both the free aneurysm wall and the portion of the wall adjacent to the intrasaccular thrombus, as illustrated in Fig. 3.

Py-Radiomics (https://pyradiomics.readthedocs.io/en/latest/) was used to retrieve RFs from the aneurysm wall and the IST (Fig. 3). One hundred thirty (130) RFs were extracted, involving 19/130 shape features, 36/130 first-order features, and 75/130 second-order features. In the end, two sets of RFs were obtained for each IA, namely, enhancement from the aneurysm wall free of IST and enhancement from the aneurysm wall next to IST.

In this study, spatial registration/correspondence between radiomics and CFD analyses was automatically achieved by using a shared (image) segmentation mask as a reference. Specificially, both the radiomic features and CFD simulations were derived from the same 3D aneurysm model, which was manually segmented from the original HR-MRI VWI images. The model was then divided into wall and lumen components for the respective analyses.

### Statistical and Visual Analysis

Spearman’s correlation values were calculated between RFs of the wall and IA parameters under the following three categories: (1) IA morphology, (2) WSS-related metrics, and (3) flow vortex core-related parameters. The correlation analysis (i.e., corr function) was conducted using the Statistics toolbox under MATLAB (Mathworks Inc., MA, USA). A subgroup analysis was conducted between RFs and hemodynamic parameters among saccular and fusiform thrombosed IAs. A strong correlation was defined as Rho > 0.7. A p-value less than 0.05 was considered statistically significant.

## Results

Forty-four thrombosed IAs were identified in our HR-MRI database, with 4/44 cases (9.1%) excluded due to flow artifacts, 2/44 (4.5%) due to poor image quality, and 1/44 (2.3%) cases due to difficulties in creating a CFD model. Thirty-seven thrombosed IAs were included for the analysis, comprising 22/37 (59.5%) fusiform and 15/37 (40.5%) saccular IAs. The most common location was the cavernous internal carotid artery (ICA) segment, observed in 16/37 cases (43.2%).

### Correlation Analysis

Table 1 shows six AWE RFs significantly correlated with aneurysm morphological features.

**Table 1:** A summary of highly correlated RFs with aneurysm morphology. GLDN.DN — Dependence Nonuniformity (DN) in gray level dependence matrix (GLDM), GLRLM.GLN — Gray Level Nonuniformity (GLN) in gray level run length matrix (GLRLM), GLSZM.SZN — Size Zone Nonuniformity (SZN) in gray level size zone matrix (GLSZM). More detailed descriptions of these AWE RF can be found in the Supplementary Materials

**Category **	**Radiomic Features **	**Spearman’s Correlation **	**Aneurysm Volume **	**P-value**	
		**Aneurysm Surface Area **			
Aneurysm wall free of IST		Total Energy	0.74	0.75	< 0.001
		GLDM.DN	0.63	0.7	< 0.001
		GLRLM.GLN	0.73	0.74	< 0.001
		GLSZM.SZN	0.7	0.7	< 0.001
Aneurysm wall next to IST		Total Energy	0.71	0.7	< 0.001
		GLRLM.GLN	0.71	0.61	< 0.001

As shown in Table 2, ten AWE RFs exhibit strong correlations with flow vortex parameters. Notably, these correlations are generally stronger (Correlation > 0.75) than those observed with morphological parameters. Additionally, we found that the NGTDN.Complexity in the enhancement of the aneurysm wall next to IST shows a strong correlation with both OSI and RRT (Table 3).

**Table 2:** A summary of highly correlated radiomic features of AWE with vortex core parameters evaluated within the aneurysm dome. GLDM.DN — Dependence Nonuniformity (DN) in gray level dependence matrix (GLDM), GLRLM.GLN — Gray Level Nonuniformity (GLN) in gray level run length matrix (GLRLM), GLSZM.SZN — Size Zone Nonuniformity (SZN) in gray level size zone matrix (GLSZM), GLRLM.RLN — Run Length Nonuniformity (RLN) in GLRLM, VV — Vortex volume, NOC — Number of (flow vortex) cores, and NOC-STD — the standard deviation of the Number of (flow vortex) cores over a cardiac cycle, which is an indicator of flow temporal stability. More detailed descriptions of these AWE RF can be found in the Supplementary Materials

Category	Radiomic Features	Spearman’s Correlation	*P*-value	
Aneurysm wall free of IST		Total Energy	VV (0.80), NOC (0.83), NOC-STD (0.73)	< 0.001
		GLDM.DN	VV(0.72)	< 0.001
		GLRLM.GLN	VV(0.84), NOC (0.75)	< 0.001
		GLSZM.SZN	VV (0.82), NOC (0.78), NOC-STD (0.70)	< 0.001
Aneurysm wall next to IST		Energy	VV (0.72), NOC (0.77), NOC-STD (0.83)	< 0.001
		Total Energy	VV (0.75), NOC (0.80), NOC-STD (0.86)	< 0.001
		GLDM.DN	VV (0.78), NOC (0.81), NOC-STD (0.82)	< 0.001
		GLRLM.RLN	VV (0.79), NOC (0.82), NOC-STD (0.83)	< 0.001
		GLRLM.GLN	VV (0.76), NOC (0.77), NOC-STD (0.73)	< 0.001
		GLSZM.SZN	VV (0.70), NOC (0.72), NOC-STD (0.74)	< 0.001

**Table 3:** A summary of highly correlated (i.e., Correlation > = 0.7) AWE RFs-lumen image markers with WSS-related parameters evaluated at the aneurysm dome. NGTDN stands for neighbouring Gray tone difference matrix. OSI and RRT stand for oscillatory shear index and relative residence time, respectively

Radiomic Feature		WSS Metric	Spearman’s Correlation	*P*-value
NGTDN.Complexity in the aneurysm wall next to IST		Average OSI	0.70	**< 0.001**
		Average RRT	0.70	**< 0.001**

Subgroup Analysis

**Fig. 4:**
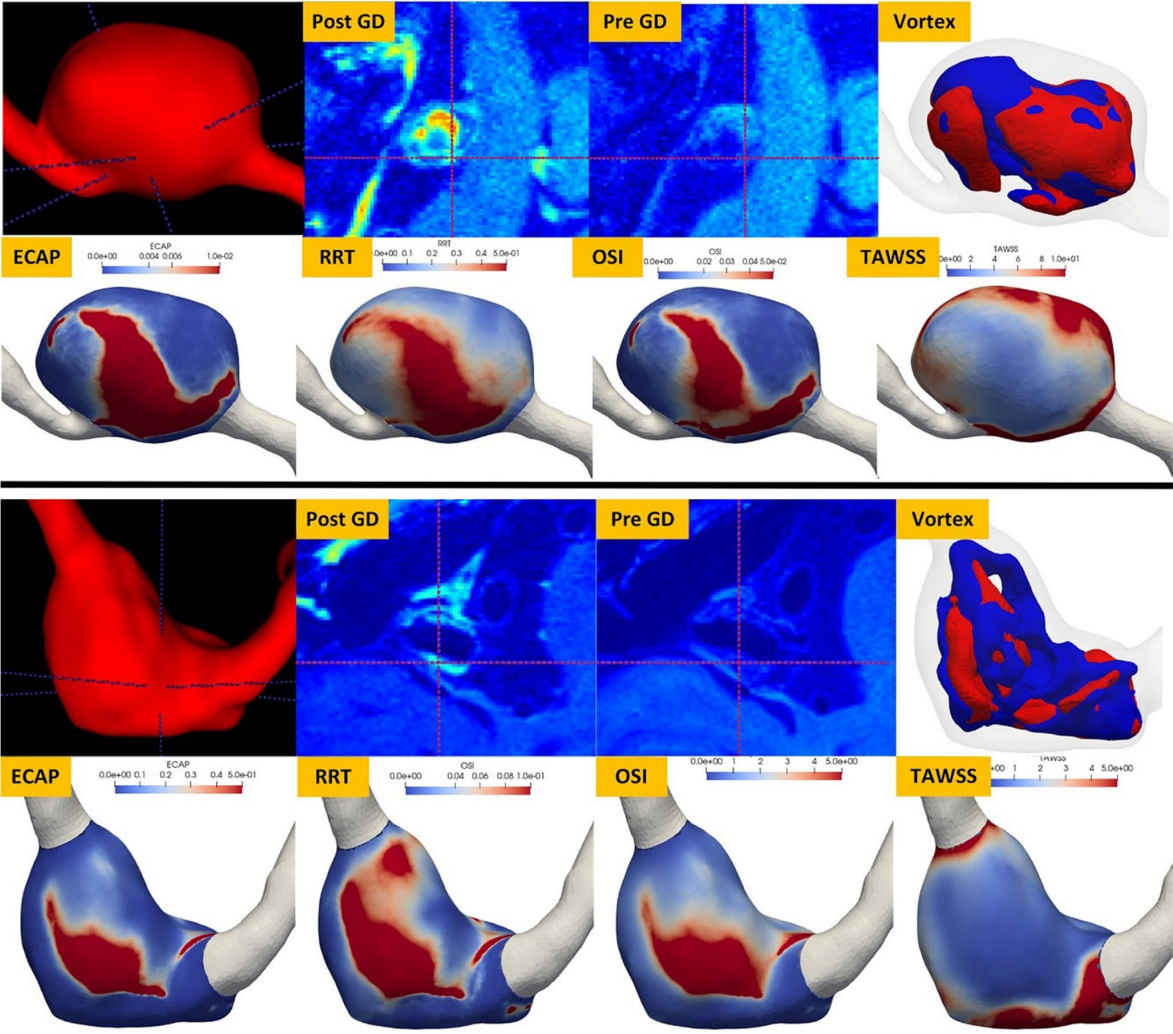
Visual assessment of the correlation between AWE distribution, vortex parameters, and WSS-derived parameters (Fig. 4). Note that the red 2D crosshair cursors on the pre- and post-Gd images are spatially co-registered with the 3D cursor position in the volumetric view. The unit used for TAWSS is Pa. OSI **—** Oscillatory Shear Index, ECAP **—** Endothelial Cell Activation Potential, RRT **—** Relative Residence Time, and TAWSS **—** Time-averaged WSS

In the subgroup analysis, Spearman’s correlations between RFs of AWE and hemodynamic parameters were calculated separately for saccular and fusiform IAs. Parameter pairs with high correlations (> = 0.7) are shown in Tables 4 and 5, respectively. Overall, a higher number of pairs of RFs of AWE and hemodynamic parameters with high correlation was identified in Saccular IAs (30 [Table 4] versus 11 [Table 5]). This discrepancy can be explained as follows. Among saccular IAs, many RFs of AWE were highly correlated with three WSS metrics, i.e., avg OSI, avg ECAP, and avg RRT. In contrast, such high correlation did not exist among fusiform IAs (15 [Table 4] vs. 0 [Table 5]). The AWE on MRI scans was visually compared with the distribution of WSS-derived parameters and vortex cores to qualitatively validate the hypothesis that AWE spatial patterns correlate strongly with gross aneurysmal hemodynamics. The results from the two sample cases in Fig. 4 strongly support the conclusions presented in previous sections.

**Table 4:** A summary of highly correlated RFs of AWE image markers with vortex core parameters and WSS metrics evaluated within the saccular IA dome. RMS — Root Mean Squared, CP — Cluster Prominence, CS — Cluster Shade, GLCM.DA — Difference Average (DA) in gray level co-occurrence matrix (GLCM), GLCM.DV — Difference Variance (DV) in gray level co-occurrence matrix (GLCM), GLDM.DN — Dependence Nonuniformity (DN) in gray level dependence matrix (GLDM), GLDM.DNN — Dependence Nonuniformity Normalized (DNN) in GLDM, GLDM.HGLE — High Gray Level Emphasis (HGLE) in GLDM, GLDM.SDHGLE– Short Dependence High Gray Level Emphasis (SDHGLE) in GLDM, GLRLM.RLN — Run Length Nonuniformity (RLN) in gray level run length matrix (GLRLM), GLRLM.HGLRE — High Gray Level Run Length Emphasis (HGLRLE) in GLRLM, GLRLM.LRHGLE — Long Run High Gray Level Emphasis (LRHGLE) in GLRLM, GLSZM.SZN **—** Size Zone Nonuniformity (SZN) in gray level size zone matrix (GLSZM), GLSZM.GLN **—** Gray Level Nonuniformity (GLN) in GLSZM, GLSZM.GLV **—** Gray Level Variance (GLV) in GLSZM, GLSZM.HGLZE **—** High Gray Level Zone Emphasis (HGLZE) in GLSZM, GLSZM.SAHGLE **—** Small Area High Gray Level Emphasis (SAHGLE) in GLSZM, NGTDN — Neighbouring Gray Tone Difference Matrix, OSI **—** Oscillatory Shear Index, ECAP **—** Endothelial Cell Activation Potential, RRT **—** Relative Residence Time, VV — Vortex volume, NOC — Number of (flow vortex) cores, and NOC-STD — the standard deviation of the Number of (flow vortex) cores over a cardiac cycle, which is an indicator of flow temporal stability. More detailed descriptions of these RF features of AWE can be found in the Supplementary Materials

**Category **	**Radiomic Features **	**Spearman’s Correlation **	**P-value**	
Aneurysm wall free of IST		Mean	Avg OSI (0.71)	0.003
		Minimum	Avg OSI (0.80), Avg ECAP (0.80), Avg RRT (0.81)	< 0.001
		RMS	Avg OSI (0.71)	< 0.001
		Total Energy	VV (0.88), NOC (0.84)	< 0.001
		CP	Avg OSI (0.78), Avg ECAP (0.74), Avg RRT (0.74)	< 0.001
		CS	Avg OSI (0.81), Avg ECAP (0.80), Avg RRT (0.80)	< 0.001
		Contrast	Avg OSI (0.86), Avg ECAP (0.82), Avg RRT (0.82)	< 0.001
		GLCM.DA	Avg OSI (0.77), Avg ECAP (0.73), Avg RRT (0.73)	0.002
		GLCM.DV	Avg OSI (0.83), Avg ECAP (0.79), Avg RRT (0.79)	< 0.001
		GLDM.DN	VV (0.91), NOC (0.84)	< 0.001
		GLDM.DNN	Avg OSI (0.72)	0.003
		GLRLM.RLN	VV (0.87), NOC (0.85)	< 0.001
		GLSZM, GLN	VV (0.86), NOC (0.84)	< 0.001
		GLSZM.GLV	Avg OSI (0.70)	0.004
		GLSZM.SZN	VV (0.84), NOC (0.85)	<=0.001
		NGTRM.Coarseness	VV (−0.75)	0.003
		NGTRM.Strength	Avg OSI (0.95), Avg ECAP (0.95), Avg RRT (0.96)	< 0.001
Aneurysm wall next to IST		GLDM.DN	NOC-STD (0.76)	0.001
		GLDM.HGLE	Avg OSI (0.83), Avg ECAP (0.81), Avg RRT (0.81)	< 0.001
		GLDM.SDHGLE	Avg OSI (0.92), Avg ECAP (0.90), Avg RRT (0.90)	< 0.001
		GLRLM.HGLRE	Avg OSI (0.83), Avg ECAP (0.81)	< 0.001
		GLRLM.LRHGLE	Avg OSI (0.79), Avg ECAP (0.77), Avg RRT (0.77)	< 0.001
		GLRLM.RLN	NOC-STD (0.77)	< 0.001
		GLRLM.SRHGLE	Avg OSI (0.84), Avg ECAP (0.82), Avg RRT (0.82)	< 0.001
		GLSZM.GLN	NOC-STD (0.71)	0.003
		GLSZM.GLV	Avg OSI (0.74), Avg ECAP (0.71), Avg RRT (0.71)	0.002–0.003
		GLSZM.HGLZE	Avg OSI (0.84), Avg ECAP (0.81), Avg RRT (0.81)	< 0.001
		GLSZM.SZN	Avg OSI (0.71)	0.003
		GLSZM.SAHGLE	Avg OSI (0.89), Avg ECAP (0.87), Avg RRT (0.87)	< 0.001
		NGTDM.Complexity	Avg OSI (0.96), Avg ECAP (0.95), Avg RRT (0.95)	< 0.001

**Table 5:** A summary of highly correlated AWE RFs with vortex core parameters and WSS metrics evaluated within the fusiform IA dome. GLDM.DN — Dependence Nonuniformity (DN) in gray level dependence matrix (GLDM), GLDM.GLN — Gray Level Nonuniformity (GLN) in GLDM, GLRLM.RLN — Run Length Nonuniformity (RLN) in gray level run length matrix (GLRLM), GLRLM.GLN — Gray Level Nonuniformity (GLN) in GLRLM, GLSZM.SZN **—** Size Zone Nonuniformity (SZN) in gray level size zone matrix (GLSZM), GLSZM.GLN **—** Gray Level Nonuniformity (GLN) in GLSZM, VV — Vortex volume, and NOC — Number of (flow vortex) cores. More detailed descriptions of these AWE RFs can be found in the Supplementary Materials

**Category **	**Radiomic Features **	**Spearman’s Correlation **	**P-value**	
Aneurysm wall free of IST		Total Energy	VV (0.82), NOC (0.84)	< 0.001
		GLSZM.GLN	VV (0.83), NOC (0.73)	< 0.001
		GLSZM.SZN	VV (0.86), NOC (0.82)	< 0.001
Aneurysm wall next to IST		Energy	VV (0.82), NOC (0.90)	< 0.001
		Total Energy	VV (0.85), NOC (0.92)	< 0.001
		GLDM.DN	VV (0.91), NOC (0.94)	< 0.001
		GLDM.GLN	VV (0.73), NOC (0.75)	< 0.001
		GLRLM.GLN	VV (0.75)	< 0.001
		GLRLM.RLN	VV (0.91), NOC (0.95)	< 0.001
		GLSZM.GLN	VV (0.89), NOC (0.90)	< 0.001
		GLSZM.SZN	VV (0.85), NOC (0.88)	< 0.001

### Qualitative examples

As demonstrated in Fig. 4, AWE in post-Gd scans shows spatial correlation with WSS distribution. Furthermore, vortex core analysis reveals that regions with low vortex core overlap correspond to areas of high wall stress variability (e.g., elevated WSS and ECAP).

## Discussion

This study correlated RFs of AWE and gross aneurysmal hemodynamic patterns in thrombosed IAs. The overall high intensity of AWE, as determined with radiomics, was positively correlated with IAs with large aneurysm surface areas and volumes (Table 1). Moreover, highly heterogeneous aneurysm walls were positively correlated with large IAs. This finding suggests that highly enhancing and heterogeneous aneurysm walls are frequently found in large thrombosed IAs. High total energy (Table 2) is associated with disturbed aneurysmal flow. When combined with a large vortex core volume and a high number of vortex core regions, these features strongly suggest highly disruptive intra-aneurysmal flow. Such hemodynamic disturbances can induce structural and functional cellular changes within the aneurysm wall, ultimately promoting thrombus formation.

Many studies have investigated the relationship between AWE and hemodynamic parameters in IAs [34–36]. For example, Owais Khan et al. analyzed 25 IAs and found that regions with lower WSS were independently associated with regions harboring AWE [37]. Similarly, Liang et al. studied 21 fusiform IAs and reported that areas exhibiting AWE were positively correlated with OSI and RRT but negatively correlated with TAWSS [38]. However, little is known of the relationship between hemodynamics and radiomics-based analysis of AWE among thrombosed IAs.

The pathogenesis of thrombosis in IAs remains a topic of ongoing debate. Krings et al. have proposed that thrombus formation may result from intramural hemorrhage, suggesting that the thrombus resides within the aneurysm wall rather than the lumen [2]. This mechanism implies an underlying dissection process rather than a purely intrasaccular event. IST has also been linked to flow stasis and specific morphological features of IAs [39]. Consequently, in many cases, it is difficult to distinguish between intramural and intrasaccular thrombus definitively. Furthermore, it remains unclear whether the low-flow hemodynamic conditions associated with IST can contribute to arterial dissection and mural thrombus formation, or whether this process leads to IST.

AWE has been associated with aneurysm wall remodeling, which can ultimately lead to aneurysm instability [40]. Interestingly, the walls of thrombosed IAs have been linked to distinct inflammatory processes. For example, Atlas et al. identified macrophages distributed throughout the aneurysm wall near the thrombus in thrombosed IAs [41]. Similarly, Schubiger et al. reported that the aneurysm walls of thrombosed IAs are vascularized by vasa vasorum, which behave similarly to the membranes seen in subdural hematomas, leading to intramural hemorrhages and subsequent aneurysm growth [3]. Therefore, exploring the correlation between AWE and hemodynamic metrics may provide valuable insights into the pathophysiology of thrombosed IAs.AWE has been reported to be associated with low WSS areas [42, 43]. Our study builds on these findings by demonstrating that AWE heterogeneity near the aneurysm wall is strongly correlated with elevated OSI and RRT (Table 3). Such AWE heterogeneity is correlated with large ECAP and higher maximum TAWSS (Spearman correlation around 0.65). This trend is considerably more prominent in saccular than fusiform IAs. Notably, stronger correlations between the AWE heterogeneity and strength (e.g., NGTDM.Complexity and NGTDM.Strength) and ECAP are shown in Table 4. Therefore, heterogeneous blood flow demarcated by elevated OSI and RRT correlated with RFs associated with heterogeneous SI might harbor inflammatory processes in the aneurysm wall of thrombosed IAs.

Fusiform aneurysms exhibit distinct pathophysiological mechanisms compared to saccular aneurysms. They are often associated with underlying vasculopathies such as atherosclerosis and arterial dissection [44–46]. In our analysis, fusiform IAs demonstrated slower and more stagnant blood flow than saccular aneurysms, potentially predisposing them to IST [47, 48]. Among saccular thrombosed IAs, RFs of the aneurysm wall adjacent to the thrombus showed stronger correlations with OSI and RRT, compared with RFs from thrombus-free regions of the wall. Similarly, in fusiform thrombosed IAs, RFs of the wall adjacent to IST were more strongly correlated with VV and NOC, relative to thrombus-free wall segments (Tables 4 and 5).

The combination of increased near-wall oscillations and elevated RRT suggests that AWE is more likely to occur in larger aneurysms characterized by extensive low-velocity recirculation zones. These zones are often associated with larger vortex core volumes and a greater number of core regions. Taken together, findings from the vortex flow analysis and WSS-derived parameters indicate that high and heterogeneous AWE are associated with large, thrombosed intracranial aneurysms. These aneurysms are frequently marked by pronounced intra-aneurysmal flow disturbances, particularly sizable low-velocity recirculation zones within the aneurysm sac.

The correlations between WSS-related parameters and AWE RFs are weaker than those between the AWE and gross hemodynamic parameters (i.e., through flow vortex core analysis). This finding can be partially related to diverse cellular changes induced by abnormal WSS distributions [49], which may not singularly increase AWE or its heterogeneity (see Figs. 2 and 3). Similarly, WSS-related parameters are less influential in predicting IAs’ rupture risk. It is important to note that WSS-related parameters are derived from the flow motion near the aneurysm wall. In contrast, flow vortex parameters capture gross swirling blood flow patterns within the aneurysmal dome. Including the quantitative analysis of flow vortices benefited our understanding of IA development [32, 50], its rupture status [51], and the growth status of abdominal aortic aneurysms [52]. Biologically, recirculating flow eddies within the IA sac introduce cellular structural and functional changes; these cellular changes disturb normal physiological morphology, cell-to-cell adhesion, mechanotransduction, and cellular genetic expression [9, 53, 54]. Identifying such hemodynamic disturbances—alongside increased AWE through radiomic analysis—may enhance risk assessment for thrombosis in both saccular and fusiform aneurysms.

This study has several limitations. First, it is a retrospective, single-center study, which may limit the generalizability and reproducibility of our findings. Second, the sample size was limited since IST is a relatively rare phenomenon, mostly seen in large aneurysms. Similarly, longitudinal data were not available since most aneurysms were treated, or in some instances, patients were not imaged with the same HR-MRI protocol. Third, because 4D phase-contrast MR imaging is not routinely used for monitoring IAs’ progression, patient-specific flow boundary conditions are not available. As a result, averaged flow (rate) waveforms measured from multiple human subjects by phase-contrast MR imaging were used as the inflow boundary condition in this study. We anticipate that the simulation accuracy can be improved by “patient-specific” flow boundary conditions [55–57]. Moreover, complex intrasaccular thrombosis (e.g., spatially varying porosity, permeability, etc.) was not considered during the CFD simulations and will be considered in our future studies. Lastly, in certain cases, it is challenging to distinguish whether the thrombus is intramural or intrasaccular. Therefore, we encourage histopathological and imaging correlations of thrombosed IAs for a better understanding of this process. As a result, some dissecting aneurysms with intramural thrombosis may have been inadvertently included in this study. However, as noted by Krings et al., intramural thrombosis may represent part of the pathological continuum of IST [2]. 

## Conclusion

RFs depicting higher and heterogeneous SI throughout the aneurysm wall are predominant in thrombosed IAs, often accompanied by relatively large, low-velocity recirculation zones within the IA domes. Since AWE may be linked to degenerative vascular remodeling of the aneurysm wall, continued analysis of the spatiotemporal characteristics of hemodynamic flow patterns within aneurysms can improve the understanding of conditions leading to such degenerative vascular remodeling. Our future work may assist in differentiating IAs at high risk of rupture from those likely to remain asymptomatic.

## Supplementary Information

Below is the link to the electronic supplementary material.

ESM 1  (DOCX 608 KB)

## Data Availability

No datasets were generated or analysed during the current study.
