# Supplementary material for: Correlations between hemodynamics and radiomic features in thrombosed intracranial aneurysms
Source: Neuroradiology. 2025 Aug 18;67(9):2447–58. doi: 10.1007/s00234-025-03730-x (PMC12546522; doi:10.1007/s00234-025-03730-x)
Supplement: Supplementary file 1 — (DOCX 608 KB) [file 234_2025_3730_MOESM1_ESM.docx]

**Supplementary Materials to** Manuscript entitled “Correlations Between Hemodynamics and Radiomic Features in Thrombosed Intracranial Aneurysms”

**Section 1 - Patient Cohort: Image acquisition protocol**

As discussed in the Methods and Materials Section, T1-weighted (T1) and T1+Gadolinium (T1+Gd) sequences were acquired for all patients using the setup outlined in Table S1.

**Supplementary Table S1: 3T Siemens high-resolution magnetic resonance imaging protocol.**

|  | **SAG 3D T1 SPACE FS** |
| --- | --- |
| TR (msec) | 900 |
| TE (msec) | 15 |
| Flip angle (deg) | variable |
| Bandwidth (Hz/pixel) | 446 |
| FOV (mm) | 200 x 200 |
| Matrix (mm) | 320 x 320 |
| Voxel size (mm) | 0.6 x 0.6 x 0.6 |
| Slice thickness, mm | 0.63 |
| Turbo Factor | 52 |
| Acquisition time (min) | 3:29 |

**Section 2 – Aneurysm Geometrical Characteristics**

As discussed in **the manuscript's Methods and Materials, we first isolated the aneurysm** sac for each intracranial aneurysm (IA), as shown in Fig. S1. Then, the following morphological parameters were calculated (Table S2).


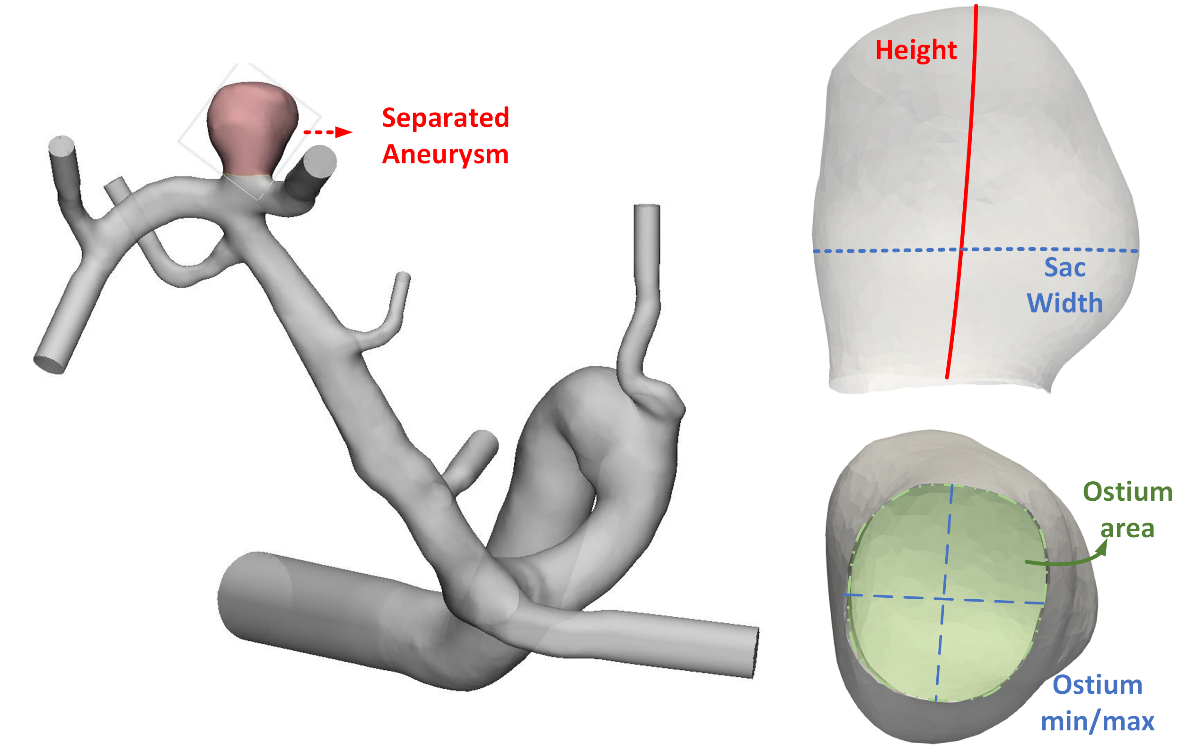


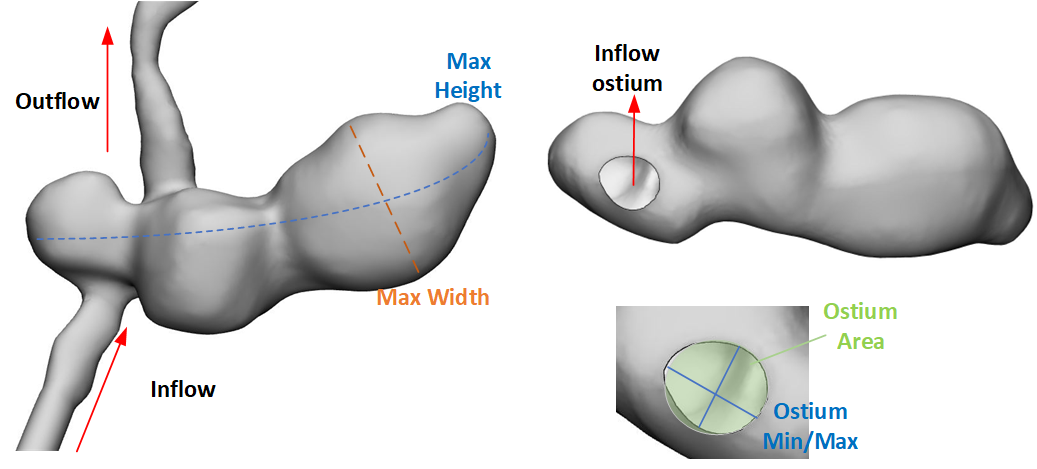


**Figure S1**: **An illustration of the geometric characteristics of typical IAs is provided.** (a) shows an isolated IA sac from the parent vessel (highlighted in red). In (b), the solid red lines represent the centerlines generated using Vascular Modeling ToolKit (VMTK), [1] where the height of the IA is determined by the length of the centerline within the aneurysm region. The blue dashed line indicates the maximum width of the IA. In (c), the green circle represents a 2D cutting plane used to measure the minimum and maximum ostium diameters, and the green surface area on the cutting plane indicates the ostium area

The geometrical characteristics of the selected intracranial aneurysms (IAs) are provided in Table S2 below.

**Supplementary Table S2:** Basic geometrical information of 37 thrombosed IAs investigated in this study

| Height (mm) | Surface Area (mm^2^) | Volume (mm^3^) | Ostium Area (mm^2^) | Aspect  Ratio | Vol/Ostium  (mm) |
| --- | --- | --- | --- | --- | --- |
| 24.1 $\pm$ 10.8 | 1527.8 $\pm$ 1255.5 | 6360.1 $\pm$ 7654.9 | 37.6 $\pm$ 27.39 | 4.0 $\pm$ 1.5 | 165.2 $\pm$ 166.6 |

**Section 3 – Radiomics Analysis of AWE**

Pyradiomics package[2] was used to compute velocity-informatics parameters in the third step. A complete list of radiomics features (RFs) can be found on the Pyradiomics website (<https://pyradiomics.readthedocs.io/en/latest/>). Most relevant RF parameters are listed below for completeness, and their definitions can be found from Pyradiomics’ documentation.

The following RF parameters were used in this study.

**First-Order Statistics:**

- Energy
- Total Eenergy
- Mean
- Minimum
- Root Mean Squared (RMS)
- Kurtosis

**Second-Order Statistics:**

A **Gray Level Co-occurrence Matrix (GLCM)** quantifies the spatial relationship between pairs of connected voxels based on their intensity values. The following parameters were used in this study.

- GLCM.CP — Cluster Prominence (CP) in GLCM,
- GLCM.CS — Cluster Shade (CS) in GLCM,
- GLCM.DA — Difference Average (DA) in GLCM,
- GLCM.DV — Difference Variance (DV) in GLCM

A **Gray Level Dependence Matrix** (GLDM) quantifies gray level dependencies in an image. A gray level dependency is defined as the number of connected voxels within a distance of $\delta$ that are dependent on the center voxel.

- GLDM.DN — Dependence Nonuniformity (DN) in GLDM
- GLDM.DNN — Dependence Nonuniformity Normalized (DNN) in GLDMGLDM.GLN — Gray Level Nonuniformity (GLN) in GLDM
- GLDM.HGLE — High Gray Level Emphasis (HGLE) in GLDM,
- GLDM.SDHGLE – Short Dependence High Gray Level Emphasis (SDHGLE) in GLDM,

**A Gray Level** **Run Length Matrix (GLRLM) is** computed based on the number of connected voxels with the same intensity. GLRLM is characterized by an angle between pairs of voxels, $\theta$.

- GLRLM.GLN — Gray Level Nonuniformity (GLN) in GLRLM
- GLRLM.RLN — Run Length Nonuniformity (RLN) in GLRLM
- GLRLM.HGLRE — High Gray Level Run Length Emphasis (HGLRLE) in GLRLM
- GLRLM.LRHGLE — Long Run High Gray Level Emphasis (LRHGLE) in GLRLM
- GLRLM.SDHGLE – Short Dependence High Gray Level Emphasis (SDHGLE) in GLRLM,

**The Gray Level** **Size Zone Matrix (GLSZM)** quantifies intensity zones in an image. A zone is defined as some connected voxels with the same intensity level.

- GLSZM.SZN — Size Zone Nonuniformity (SZN) in GLSZM
- GLSZM.GLN — Gray Level Nonuniformity (GLN) in GLSZM
- GLSZM.GLV — Gray Level Variance (GLV) in GLSZM
- GLSZM.HGLZE — High Gray Level Zone Emphasis (HGLZE) in GLSZM
- GLSZM.SAHGLE — Small Area High Gray Level Emphasis (SAHGLE) in GLSZM

A **Neighbouring Gray Tone Difference Matrix** quantifies the difference between a gray value and the average gray value of its neighbours within a distance of δ.

- NGTDN.Complexity — Complexity in Neighbouring Gray Tone Difference Matrix (NGTDN)
- NGTDN.Coarseness — Coarseness in Neighbouring Gray Tone Difference Matrix (NGTDN)
- NGTDN.Complexity — Strength in Neighbouring Gray Tone Difference Matrix (NGTDN)

Py-Radiomics was used to retrieve RFs from these components (Figure 1). A total of 130 RFs were extracted involving 19/130 shape features, 36/130 first order features and 75/130 second order features.[3] An open-source statistical analysis package, R (version 4.3.3), was used for statistical analysis. Due to the exploratory nature of this study, p-values <0.05 were considered statistically significant. A total of 6 RFs were significantly different between AWE-Lumen and AWE-Lumen + Thrombosis categories, as shown in Table S3 below.

**Supplementary Table S3: Comparison of radioomics features between AWE-Lumen and AWE-Lumen + Thrombosis categories**

| **Radiomics** | **Feature** | **P - value** |
| --- | --- | --- |
| First order | Kurtosis | **0.01** |
| GLDM | Dependence Non-Uniformity (DN) | **0.03** |
| GLSZM | Gray Level Non-Uniformity (GLN) | **0.001** |
| GLRLM | Run Length Non-Uniformity (RLN) | **0.02** |
| NGTDM | Busyness | **0.02** |
|  | Coarseness | **0.001** |

**Section 4 - Vortex core analysis**

A published computational approach [4] utilizing informational entropy was employed to determine the spatially varying direction of the velocity field for identifying flow vortices. Specifically, the 3D angular space of the velocity field was first partitioned into 360 equal-area bins, forming conical regions that connect the center of a unit sphere to surface patches. Each velocity vector was assigned to a corresponding patch if it fell within the associated cone. To evaluate the local flow direction $x\in\{x_{1},x_{2},x_{3},\ldots,x_{n}\}$ within the velocity field $X$, the probability $p\left( x_{i} \right)$ of each direction was computed and used to determine Shannon’s entropy.

$$H\left( X \right)= -\sum_{x_{i}\in X} p\left( x_{i} \right){log}_{2}p\left( x_{i} \right)$$

$$NE\left( X \right)= \frac{H(X)}{{log}_{2}(N)}$$

Since normalized entropy (NE) cannot distinguish vortices from Brownian motion, the $\lambda_{2}$​ method was incorporated. The velocity gradient tensor was decomposed into strain-rate ($S$) and spin ($\Omega$) components. Vortex cores were identified where $S^{2}+\Omega^{2}$ had two negative eigenvalues ($\lambda_{1}>\lambda_{2}>\lambda_{3}$​). The dot product between the velocity vector and eigenvector measured directional alignment, with 0 indicating co-alignment and 1 indicating orthogonality.

$$S= \frac{\nabla\vec{v}+{\nabla\vec{v}}^{T}}{2}, \Omega= \frac{\nabla\vec{v}-{\nabla\vec{v}}^{T}}{2}$$

To refine vortex detection, this alignment factor $du(\theta)$ was multiplied by NE, reducing false positives from Brownian motion.

$$du\left( \theta\right)=\left\| \vec{v} \right\|\cdot\lambda_{2}$$

This combined method (CM) was applied across all voxels in the IA dome for each cardiac cycle. Vortex regions were extracted using the marching cubes algorithm, with a threshold CM>0.3. Small regions (<0.5 mm³) were excluded to prevent misidentification.

$$CM\left( X \right)=NE(X)\times du\left( \theta\right)$$

**Section 5 – A list of hemodynamic parameters**

- OSI — Oscillatory Shear Index
- ECAP — Endothelial Cell Activation Potential
- RRT — Relative Residence Time
- VV — Vortex volume
- NOC — Number of (flow vortex) cores
- NOC-STD — the standard deviation of the Number of (flow vortex) cores over a cardiac cycle, which is an indicator of flow temporal stability.

**Section 6 – Hemodynamics versus IA morphology**

| **Supplementary Table S4:** A summary of highly correlated hemodynamic parameters with IAs’ geometric metrics. Acronyms are explained above in S2 and S5. | | | | | | |
| --- | --- | --- | --- | --- | --- | --- |
| **Category** | |  | **Geometric Features** | | **Spearman’s Correlation** | **P-value** |
| **Saccular** | | | | Surface Area | VV (0.83), NOC (0.86) | **<=0.001** |
|  |  |  |  | Volume | VV (0.84), NOC (0.90) | **<0.001** |
|  |  |  |  | Aspect Ratio | Avg ECAP (0.78) | **<0.001** |
|  |  |  |  | Vol/Ostium | VV (0.73), Avg ECAP (0.88), Avg RRT (0.74) | **<0.001** |
| **Fusiform** | | | | Surface Area | VV (0.86), NOC (0.86) | **<0.001** |
|  |  |  |  | Volume | VV (0.87), NOC (0.88) | **<0.001** |
|  |  |  |  | Height | VV (0.78), NOC (0.81) | **<0.001** |
|  |  |  |  | Aspect Ratio | VV (0.66), NOC (0.68) | **<0.001** |
|  |  |  |  | Vol./Ostium | VV (0.88), NOC (0.89) | **<0.001** |
|  | **Saccular + Fusiform** | | | Surface Area | VV (0.83), NOC (0.85) | **<0.001** |
|  |  |  |  | Volume | VV (0.83), NOC (0.87) | **<0.001** |
|  |  |  |  | Height | VV (0.77), NOC (0.77) | **<0.001** |
|  |  |  |  | Aspect Ratio | VV (0.60) | **<0.001** |

**Reference**

[1] M. Piccinelli, A. Veneziani, D. A. Steinman, A. Remuzzi, and L. Antiga (2009) A Framework for Geometric Analysis of Vascular Structures: Application to Cerebral Aneurysms. IEEE Transactions on Medical Imaging*.*  28: 1141-1155 doi: 10.1109/TMI.2009.2021652.

[2] J. J. M. van Griethuysen *et al.* (2017) Computational Radiomics System to Decode the Radiographic Phenotype. Cancer Research*.*  77: e104-e107 doi: 10.1158/0008-5472.Can-17-0339.

[3] A. Zwanenburg *et al.* (2020) The Image Biomarker Standardization Initiative: Standardized Quantitative Radiomics for High-Throughput Image-based Phenotyping. (in eng), Radiology*.*  295: 328-338 doi: 10.1148/radiol.2020191145.

[4] K. Sunderland, Q. Huang, C. Strother, and J. Jiang (2019) Two Closely Spaced Aneurysms of the Supraclinoid Internal Carotid Artery: How Does One Influence the Other? Journal of Biomechanical Engineering*.*  141: doi: 10.1115/1.4043868.
